# Preventive Role of Cocoa-Enriched Extract Against Neuroinflammation in Mice

**DOI:** 10.3390/neurolint17040047

**Published:** 2025-03-24

**Authors:** Ivan Carrera, Lola Corzo, Olaia Martínez-Iglesias, Vinogran Naidoo, Ramón Cacabelos

**Affiliations:** EuroEspes Biomedical Research Center, International Center of Neuroscience and Genomic Medicine, 15165 Bergondo, Corunna, Spain; analisis@euroespes.com (L.C.); epigenetica@euroespes.com (O.M.-I.); neurociencias@euroespes.com (V.N.); rcacabelos@euroespes.com (R.C.)

**Keywords:** neuroinflammation, cocoa, hypo-vitaminosis, animal model, polyphenols

## Abstract

Background: Chronic aberrant inflammation is a crucial step in mediating cerebrovascular and neurodegenerative pathologies, including Alzheimer’s and Parkinson’s disease. Due to their exceptional antioxidant properties and ability to alter imbalance metabolism and reactive inflammation response, cocoa-derived flavanols are being investigated as potential bioactive substances to modulate and reverse these inflammation-associated disorders. Objective: The present study will focus on the possible beneficial effects of cocoa-derived extract, enhanced with other bioactive phytochemicals such as spirulina and pineapple, on selected biomarkers of the inflammatory, metabolic, and neurodegenerative processes. Methods: A mice model of inflammation was treated with cocoa-derived extract cocktail, and biomolecular data was obtained by performing immunohistochemical and biochemical analysis. Results: Results show that the cocoa-derived extract mitigates the neuroinflammatory processes triggered (decreased expression of macrophage CD11b) and prevents the escalade of subsequent neurodegeneration pathologies. Conclusions: The results based on hypo-vitaminosis, neuroinflammation, and inmunoreactive analysis suggest that cocoa-derived extract is a powerful bioproduct for ameliorating neuroinflammatory processes that mediate metabolic and cerebrovascular diseases.

## 1. Introduction

The World Health Organization recognizes that nutrition is one of the pillars of health and development. For people of all ages, nutrition better allows them to strengthen the immune system, contract fewer diseases, and enjoy more robust health. This is a classic concept that relates an adequate diet with a correct nutritional status, a good functioning of the individual, and better recovery from diseases [1]. It is well established that poor diet plays an active role in exacerbating many chronic inflammatory diseases. Our understanding of how the immune system drives chronic inflammation and disease pathogenesis has evolved in recent years [2]. Chronic inflammation is a central process involved in a high number of metabolic disorders (e.g., obesity, metabolic syndrome, diabetes, dyslipidemia, etc.), including neurodegenerative (Alzheimer), malignant diseases, and autoimmune diseases. In most, if not all, chronic inflammatory conditions, there is an extensively failed resolution of inflammation with high influx of leukocytes, which in their effort to resolve inflammation stimulate the synthesis of pro-inflammatory molecules and establish a highly inflammatory micro-environment, leading to extensive fibrosis and tissue damage [3].

It is well known that cocoa, as well as tea, coffee, vegetables and fruits, are rich in polyphenols. This is a complex group of compounds that gained significant attention due to their antioxidative properties, thus promoting potential beneficial effects on human health [4,5] through which the content of low molecular weight flavanols, such as epicatechin, is considered of special importance. *Theobromacacao* L., an evergreen cacao tree, is recognized for its unroasted beans, which are called cocoa. Its effects on behavior modulation mainly affecting mood, hunger, and relaxation are particularly well-known. Yet, an extensive number of studies reported benefits displayed by cocoa extracts and its constituents on inflammation processes or impaired immune functions, ageing, blood pressure regulation, atherosclerosis, or cardiovascular diseases development [6,7,8]. Cocoa compounds were shown to influence platelet activation [9,10], nitric oxide (NO)-dependent activities [11,12,13], blood pressure [14], and insulin resistance [15], as well as cytokine production [16]. Some clear evidence exists about how the consumption of cocoa-rich food may reduce inflammation, mainly by lowering the activation of monocytes and neutrophils [17], although its efficacy depends on the extent of the basal inflammatory burden. Therefore, the inflammatory background in disease development, like the starting point of pro-inflammatory cytokine release and metabolite production, is a central target for preventive actions. In line with this, we aim to discuss potential interferences of cocoa antioxidants with central immunoregulatory mechanisms by focusing on pathways involved in cell-mediated immune response within the brain of animal models. Furthermore, among the most discussed effects of cocoa uptake are interferences with vascular function, platelet reactivity, and inflammatory processes, suggesting vascular-protective properties of some constituents [18,19]. Chronic inflammation is brought on by the buildup of lipids in the artery wall, inducing the vessel wall to thicken and harden, reducing its flexibility and impairing blood flow [20]. By reducing oxidation processes and/or interfering with cellular signaling pathways, a high dietary intake of antioxidant substances may reduce the risk of atherosclerosis [21,22].

Research from recent years has demonstrated that a range of phytochemicals included in fruits, vegetables, herbs, spices, and algae induce synergistic anti-inflammatory and anti-oxidant effects, which may account for the health advantages of plant-based diets [23,24,25,26]. In particular, it has been demonstrated that phytoconstituents found in spirulina (*Arthrospira maxima*) [27,28,29,30] and pineapple (*Ananas comosus*) [31,32,33] have specific beneficial antioxidant and anti-inflammatory qualities tested both in vitro and in vivo animal models as well as in clinical patients. In addition to being rich in macro- and micronutrients, they are a significant source of phenolic compounds, such as flavonoids and non-flavonoids phenolics [34,35,36], carotenoids, sterols, and stanols, as well as dietary fiber. The various biological actions of these bioactive compounds—antioxidant [37,38], immunomodulatory [39], anti-inflammatory [40,41], and anti-hypertensive [42,43]—are what have sparked interest in them. Our guiding framework was that flavonols prevent neuroinflammation and cognition deterioration parameters. Accordingly, we hypothesized that a short cocoa-enriched extract intervention, enhanced with demonstrated anti-inflammatory compounds such as spirulina and pineapple, could decrease the concentrations of key neuroinflammation markers and consequently have an impact on critical brain metabolite ratios. The overall aim of this research is to gather information about the beneficial effects of cocoa-enriched extract, supplemented with a specific phytochemical formulation (spirulina and pineapple), against neuroinflammation preventing future neurodegenerative disorders.

## 2. Materials and Methods

### 2.1. Mouse Models

13 wild-type mice (C57BL/6J) about 9 weeks old were treated with a poor diet (2 g/day of commercial feed) or with a poor diet and the treatment extract ad libitum (Table 1). For this study, 5 mice were used for the control group and 8 for the treatment group (Table 1). Mice were housed in a room with controlled temperature (20–21 °C), humidity (40–50%), and lighting (12 h light/dark cycle) and were supplied with water ad libitum. After 5 weeks of treatment, mice were euthanized, and blood and hippocampal samples were collected and analyzed. All experimental mice procedures were conformed to the guidelines established by the European Communities Council Directive (86/609/EEC), the EU Directive 2010/63/EU, and the Spanish Royal Decree 1201/2005 for animal experimentation and were approved by the Ethical Committee of the EuroEspes Biotechnology Research Centre (Permit number: EE/2023-08, 22 August 2023).

### 2.2. Biochemical Characterization of Cocoa-Enriched Extract

The cocoa-enriched extract was obtained from combining high nutritional bioproducts such as fruit (pineapple, *Ananas comosus*) and algae (spirulina, *Arthrospira maxima*) with cacao powder (*Theobroma cacao*). According to the provider (Bulk Powder Co., batch:023-2245, Colchester, Essex (GB)), the organic cacao powder was obtained from raw cacao beans that are harvested by hand in Peru, fermented to reduce bitterness, and extracted to be broken into ‘nibs’ and ground into cacao paste, meaning that all the nutritional value of the cacao is preserved. The final combined extract was obtained by using non-denaturing biotechnological methods (Patent ID: P202230047/ES2547.5) such as the lyophilization under controlled conditions of temperature, pressure, and grinding. For more details on nutrition analysis, see Supplementary Data. Treatment preparation: The organic cocoa enriched extract was integrated into the diet as pellet biscuits elaborated in our laboratory by adding 50% of powder treatment (70% cocoa, 15% spirulina, and 15% pineapple in a 100 g of final bulk) using diet wheat as the main flour and adding 10% (*w*/*w*) MilliQ-purified water for pelleting, and then drying the pellets at 34 °C overnight. (For the principle nutrient composition of the treatment diet, see Supplementary Materials.)

### 2.3. Blood Sample Preparation

At the end of the experimental period, blood collection was performed by cardiac puncture after deep anesthesia of the mouse. Commercial Greiner Bio-One (Kremsmünster, Austria) brand tubes were used, some without anticoagulant and others with EDTA K3 as anticoagulant. The intact, unprocessed EDTA tube was used to count blood cells (LEU, RBC, PLQ), hemoglobin (HGB), hematocrit (HTO), and erythrocyte indices using the MINDRAY BC-5380 hematology analyzer. After performing the blood counts, the EDTA tubes were centrifuged at 4000 rpm 4 °C for 10 min to collect the plasma, stored at −80 °C until the analysis of vitamin B9 (folate). For the determination of vitamin B9 (folate), a commercial ELISA kit from Elabscience (Houston, TX, USA) was used with absorbance reading at 450 nm in the EPOCH reader (Biotek Instruments, Winooski, VT, USA). The tubes without anticoagulant were centrifuged at 4000 rpm for 10 min to separate the serum from the cell layer, after allowing them to clot for 20 min in an upright position after extraction. The serum was stored at −80 °C until the moment of analysis of the following parameters: C-reactive protein (CRP) using turbidimetric technique in the Cobas Mira Plus biochemical analyzer (Roche Diagnostics, Basel, Switzerland) and vitamins B6 and B12 using commercial kits ELISA from Elabscience (USA) and DRG International (Springfield, NJ, USA), respectively. Both reactions were read by absorbance at 450 nm in the EPOCH reader (Biotek Instruments, USA).

### 2.4. Brain Sample Preparation

Animals under anesthesia were transcardially perfused with 0.9% NaCl followed by 4% paraformaldehyde (PFA, Cat. #43368 Alfa Aesar™, Waltham, MA, USA). Mice brains were removed and placed into 4% PFA for 48 h, then immersed in 0.1 M phosphate buffer (PB, pH 7.4) for 12 h, cryoprotected with 30% sucose in PB, embedded in optimal cutting temperature (OCT) compound (Tissue Tek, Torrance, CA, USA), and frozen with liquid nitrogen-cooled isopentane. The left half-brain was dissected to obtain the hippocampal region for gene expression analysis, and the right half-brain was cut on a cryostat as parallel series of transverse sections (18 μm-thick). Sections were then mounted on Superfrost Plus slides (Menzel Glasser, Madison, WI, USA), and stored at room temperature for histopathological analysis.

### 2.5. Immunohistochemistry

Immunohistochemical hallmarks were analyzed as previously published [44]. In summary, mice brain section slices were incubated overnight with the primary antibody against the neuron-specific protein NeuN (1:1000; MAB-377, Millipore), and detected using the Alexa Fluor-488-tagged secondary antibody (Thermo Fisher Scientific, Waltham, MA, USA). The specificity of the fluorescent immunostaining for each antibody was confirmed by omission of the primary antibody. Then, the slices were counterstained with DAPI (Vector Laboratories, Newark, CA, USA).

### 2.6. Imaging

Fluorescence signals were captured with a Leica DM6-B upright microscope (Leica Microsystems, Buffalo Grove, IL, USA) and Leica Application Suite X (LAS X) v2.4 software. The mean density among the triplicates of immunofluorescence cell markers relative to the background in each brain section image was quantified using the area/pixel analysis software (Pixcavator v4.2).

### 2.7. Statistical Analysis

Statistical analysis was performed with SPSS v. 11.0 (SPSS, Inc., Chicago, IL, USA). Previously, a homogeneity of variance test (Levene’s test) was performed to determine whether the sample was parametric or not. Based on this criterion, the means of both groups (extract group/control group) were compared with their standard deviations/errors using the *t*-test analysis for related samples. Statistical significance was determined from P equal to or less than 0.05 (* *p* < 0.05).

## 3. Results

### 3.1. Effects of the Cocoa-Enriched Extract on Inflammatory Response

Data of all the biochemical and hematological parameters carried out on the experimental groups are detailed in Table 2. This analysis was crucial to address the effectiveness of the cocoa-enriched extract on immune, energetic, and anemic status according to hematological data; hypo-vitaminosis caused by a poor diet by measuring the levels of vitamins B6, B9, and B12; and the anti-inflammatory effect indicated by the determination of C-reactive protein (CRP) levels, a universal and sensitive marker of inflammation (Table 2).

#### 3.1.1. Benefits on the Nutritional and Vitamin Status Associated with Inflammation Process

**Figure 1:**
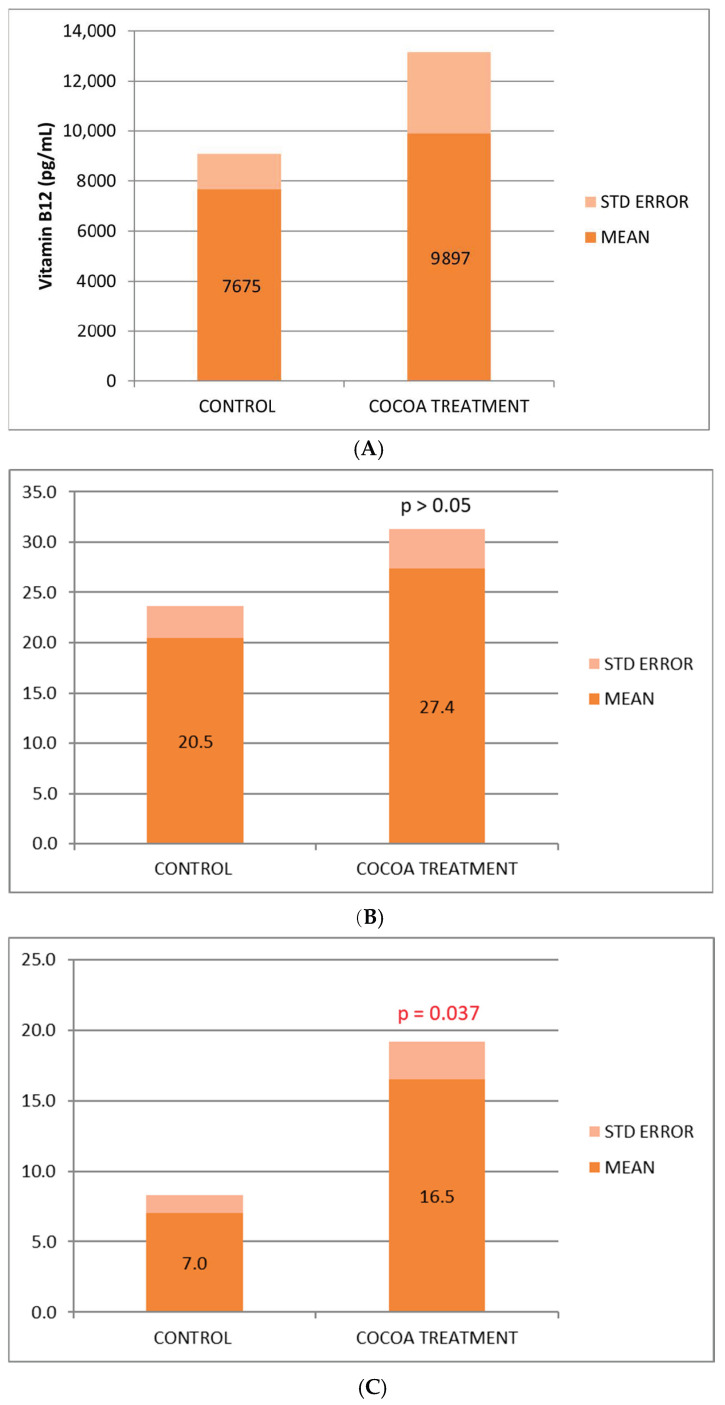
(**A**) Biochemical relative values of Vitamin B12. (**B**) Biochemical relative values of Vitamin B9. (**C**) Biochemical relative values of Vitamin B6.

We observed a beneficial effect of the cocoa-derived compound added to the diet, which increases the serum concentration of the 3 vitamins (B6, B9, and B12) analyzed (Figure 1A–C), being statistically significant for vitamin B6, in the control of inflammatory process. The vitamin increase observed in the treated group could reflect in part the anti-inflammatory, nutritional, and immunological benefits associated with this bioproduct.

#### 3.1.2. Anti-Inflammatory Effect of the Cocoa-Enriched Extract in Mice

**Figure 2:**
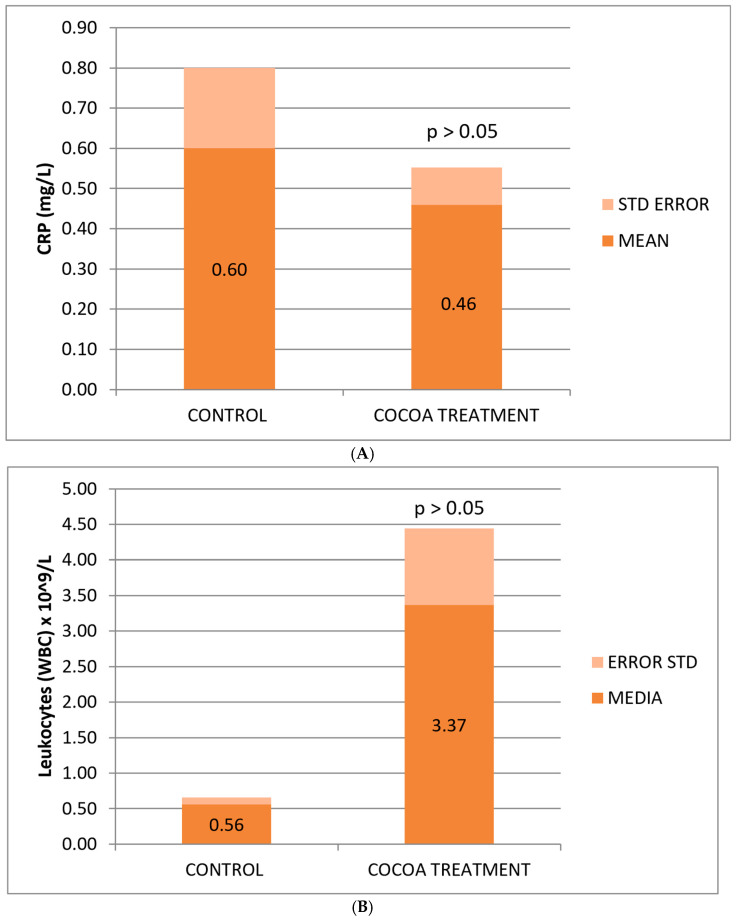

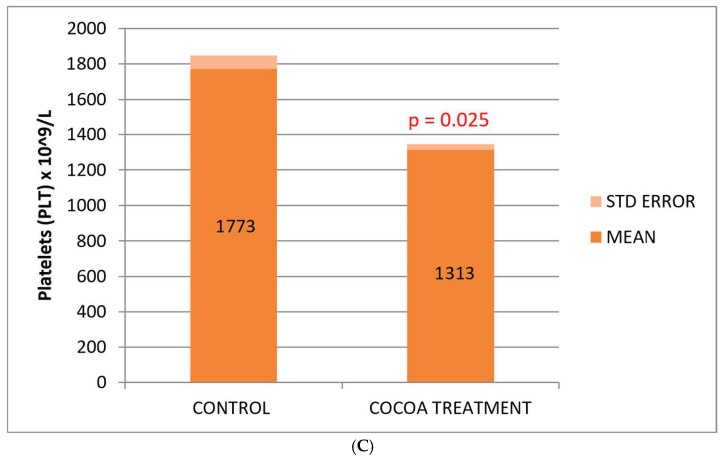
Biochemical and hematological values of CRP (**A**), leukocytes (**B**), and platelets (**C**) related to the anti-inflammatory response. Cocoa-enriched extract showed an increase in the leukocyte cell density, and slightly reduced the levels of platelets and CRP biomarker when ompared with control group.

The reduction in CRP concentration observed in treated mice was not statistically significant, possibly due to the small sample size, yet it shows a change in the anti-inflammatory effect levels of the bioproduct added to the animals’ diet (Figure 2A).

Mice fed with the cocoa-enriched extract showed an increase in the leukocyte cell count compared to those fed only with their usual diet (Figure 2B), which demonstrates the capacity of this compound to stimulate the cellular immune system of animals useful for fighting infections, immune-depression, or other associated pathologies. The difference is not statistically significant, possibly due to the high deviation of values between mice in the treated group.

No significant differences were observed between the control and treated groups with respect to the classic biomarkers of anemia (HGB, RBC, and HCT), although a slight decrease in these parameters was observed in the group of treated mice (Table 2). However, we observed a significant decrease in the platelet count compared to the control group (Figure 2C).

### 3.2. Preventive Neuroinflammation Effect of the Cocoa-Enriched Extract Diet on Mice

**Figure 3:**
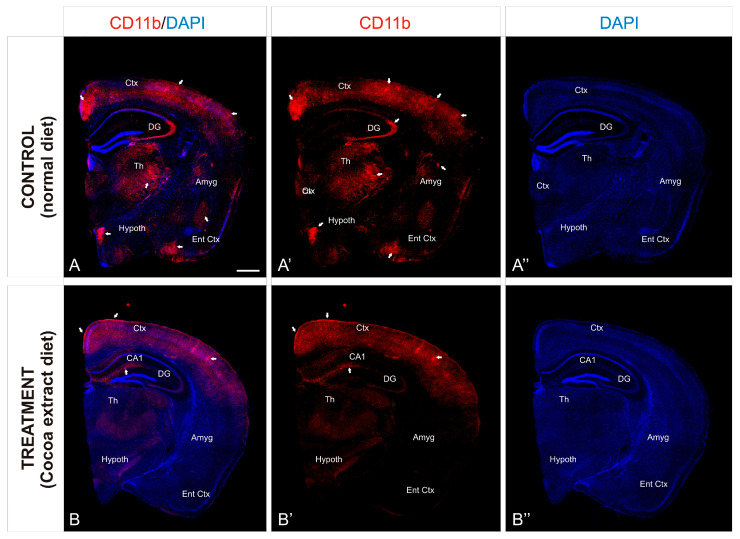
Cocoa-enriched extract is protective against neuroinflammation in mice. (**A**–**B’’**): Transverse slices of half-brain section from mice were immunostained with antibody against CD11b (red). Nuclei were counterstained with DAPI (blue). Images were processed for maximal intensity projection. The white arrows point to the most intense immunoreactive regions within the brain, mainly observed in the control non-treated mice brains (**A**–**A’’**). Scale bar: 100 μm. Abbreviations: Amyg, Amygdala; Ctx. Cortex; DAPI, neuronal nuclei marker; DG, Dentate Gyrus; Ent Ctx, Entheric Cortex; Hypoth, Hypothalamus; Th, Thalamus.

To investigate the preventive neuroinflammation effect of the cocoa-enriched extract diet on the current mice model, we performed an immunofluorescence staining analysis using antibody against CD11b as a key marker for inflammation (Figure 2). In mice brain, expression of CD11b in leukocytes increased significantly during the inflammation induction process (Figure 3A). In mice treated with the cocoa-enriched extract diet (Figure 3B), immunofluorescence expression pattern of CD11b was notably reduced and similar to healthy mice. However, untreated control mice brain showed that intermediate inflammation expression of CD11b was located in the entorhinal cortex and amygdala regions, while intense inflammation expression of CD11b was observed in frontal cortex layers, dentate gyrus, thalamus, and hypothalamus (Figure 3A,A’).

**Figure 4:**
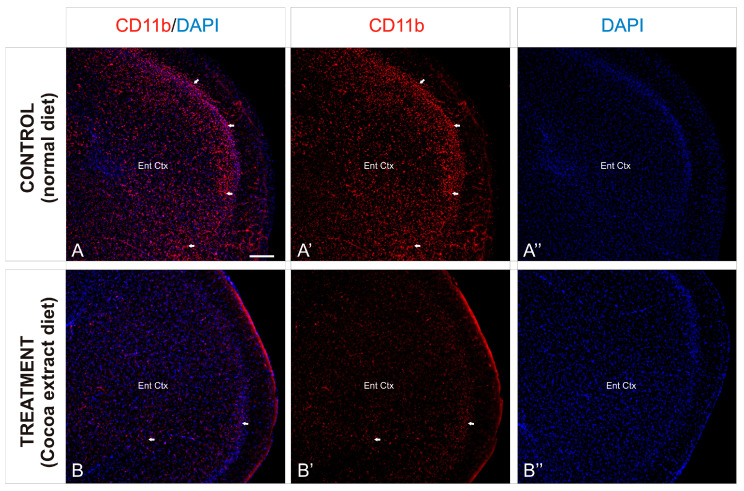
Cortical inflammation differences between mice groups. (**A**–**B’’**): Transverse slices of entorhinal cortex detail from mice were immunostained with antibody against CD11b (red). Nuclei were counterstained with DAPI (blue). Images were processed for maximal intensity projection. The white arrows show the most intense immunoreactive regions within the brain, mainly observed in the control non-treated mice brains (**A**–**A’’**). Scale bar: 100 μm.

In order to observe the different inflammation patterns between the experimental mice groups, detail of the entorhinal cortex was analyzed (Figure 4) and the immunoreactivity against CD11b was analyzed. As described above, the entorhinal cortex of the non-treated mice showed an intense distribution of immunoreactive CD11b cells, mainly gathered in the outer layers of the cortex (Figure 4A,A’), although a profuse distribution of this marker was observed throughout the entire region. In contrast, the same cortical region of the treated mice showed a scarce presence of immunoreactivity against CD11b, with no particular cluster or accumulation site within the analyzed region (Figure 4B,B’).

## 4. Discussion

Inflammation, when regulated, is a normal immune response characterized by increased blood flow, capillary vasodilation, leukocyte infiltration, and the local production of inflammatory mediators in response to cell damage. However, the unregulated inflammatory response can become chronic and contribute to the perpetuation and progression of the disease. High levels of mediators are destructive and contribute to clinical symptoms, while a rigorous biomarker screening combining multiple inflammatory markers is essential to obtain an informative assessment of the ongoing inflammation process [45,46,47]. Currently, the most widely used test in clinical practice to monitor inflammation is C-reactive protein (CRP), a circulating plasma protein that increases its levels in response to inflammation (acute phase protein). It is synthesized primarily in the liver, in response to IL-6 [48,49], and is known to play an important role in regulating the intensity and extent of acute inflammation. Therefore, CRP allows the assessment of the extent and severity of inflammation, as well as the monitoring and determination of the prognosis of subjects with long inflammatory processes [50,51].

Healthy eating habits have been associated with low levels of markers of inflammation. Several dietary components including long-chain omega-3 fatty acids, antioxidant vitamins, plant flavonoids, prebiotics, and probiotics have the potential to modulate the predisposition to chronic inflammatory conditions and may have a key role in homeostasis recovery therapy. Over the past few years, other marine nutraceuticals have been shown to improve health [52]; in particular, they improve immune response [53] by regulating lipid metabolism [54] or maintaining hormonal and bone homeostasis [55]. These components act through a variety of mechanisms, including decreasing the production of inflammatory mediators through effects on cell signaling and gene expression (omega-3 fatty acids, vitamin E, plant flavonoids), reducing the production of harmful oxidants (vitamin E and other antioxidants), and promoting gut barrier function and anti-inflammatory responses (prebiotics and probiotics) [54]. In the present study, the reduction in CRP concentration observed in treated mice reflects a possible anti-inflammatory power of the cocoa-enriched extract added to the animals’ diet.

B vitamins belong to a vitamin complex called water-soluble, which means that the body cannot store them, and they must be ingested daily through food, as they are basic for the body. Its functions are so essential that a small deficit can lead to chronic and irreversible disorders. Vitamin B deficiency can lead to several dysregulations, including anemia, muscle weakness, skin alterations, irritability, and fatigue. All of these are involved in gut immune regulation (e.g., by mediating lymphocyte migration to the gut in the case of vitamin B6, while folate is essential for the survival of regulatory T cells in the small intestine, and human gut microbes use vitamin B12 as a cofactor for metabolic pathways), thus supporting the intestinal barrier. Numerous studies demonstrate the beneficial effect of B vitamins (especially B6, B9, and B12) on the prevention and improvement of cognitive decline. Vitamin B6 maintains or enhances the cytotoxic activity of NK cells, while being necessary in the endogenous synthesis and metabolism of amino acids, building cytokines and helping to regulate inflammation (lower rates of inflammation are the outcome of higher levels of the active form). On the other hand, vitamin B12 can act as an immunomodulator for cellular immunity, and also has effects on cytotoxic cells (e.g., NK cells, cytotoxic T cells), while being crucial for metabolism and red blood cells formation, as well as improving the functioning of the central nervous system [56]. Clinical treatment with vitamin B complex has been shown to decrease pro-inflammatory expression and increase the expression of anti-inflammatory cytokines, thereby contributing to the resolution of neuroinflammation [57]. Furthermore, studies have shown that neuroinflammation is linked to depression and may explain the intricate relationship between depression and cognition in older adults. Conversely, anti-inflammatory therapies have been shown to enhance cognition [58]. A possible mechanism involved is the mobilization of these vitamins to sites of inflammation where it can serve as a cofactor in pathways that produce metabolites with immunomodulatory effects [59,60,61].

A study examined 168 older people who experience certain levels of mental decline, known as mild cognitive impairment. This condition, marked by mild memory lapses and language problems, goes beyond what might be explained as normal aging and may be a precursor to Alzheimer’s disease and other forms of dementia. After two years of taking vitamins [folic acid (B9), B6, and B12] at high doses orally, the authors found that brain shrinkage had decreased by 30% less in the treated group compared to the control group that had taken the placebo [62,63]. After 60 years of age, the brain shrinks at a rate of 0.5% annually; however, minor cognitive impairment causes the brain to shrink twice as quickly, up to 2.5% annually in Alzheimer’s disease patients. Three years later, another randomized controlled study in elderly subjects at increased risk of dementia (mild cognitive impairment according to the 2004 Petersen criteria), confirmed that treatment for 2 years at high doses of vitamin B (folic acid 0.8 mg, vitamin B6 20 mg, vitamin B12 0.5 mg) slowed the contraction of the entire brain volume. In this case, vitamin B treatment reduced brain atrophy in those gray matter regions specifically vulnerable to the AD process by up to seven times, including the medial temporal lobe [60]. In this study we observed a beneficial effect of the compound provided to the diet, which increases the serum concentration of the 3 vitamins under study (B6, B9, and B12), being statistically significant for vitamin B6, the one with the greatest involvement in the control of the inflammatory process. This increase in vitamins reflected in the group of treated mice could be part of the anti-inflammatory, nutritional, and immunological effect found in this bioproduct.

The inflammatory process begins when leukocytes that travel through the center of the bloodstream attach to the endothelium, attracted to molecules and other inflammatory cells, and migrate through the endothelial cells to the inflammatory focus. Among the main features of inflammation are increased blood flow, increased capillary and venous permeability, and mobilization of inflammatory cells out of the bloodstream [64]. Based on the results observed, we can think that the increase in cellular immune response observed with respect to the basal group fed with a poor diet could be due, in addition to their own stimulation, to the lower migration of inflammatory cells from the blood to the foci of inflammation. Taking this assumption into account, it could be another important indicator of the anti-inflammatory power of this bioproduct. Moreover, in this study of low-vitamin-diet mice model, we also find a decrease in PLT count from cocoa-treated mice. One of the most common causes of increased platelets is iron deficiency anemia or an inflammatory process. High platelets can be caused by a decrease in iron deposits in the blood, which is known as iron deficiency, or by iron deficiency anemia, that is, when a persistent lack of iron is the cause of the anemia. Taking into account that we did not observe an increment in the most commonly used indicators of anemia (HGB, RBC, and HCT), the anti-inflammatory hypothesis seems to be the most likely. The fact that plateletcrit (PCT) and mean platelet volume (MPV) increase after the treatment could indicate the presence of platelet accumulations or an individual increase in platelet size after the cocoa-enriched extract intake.

The defensive process known as neuroinflammation works to shield the central nervous system (CNS) from damage and viral assaults. Most of the time, it’s a helpful process that ends when the danger is removed, and homeostasis is restored [65]. According to McGeer and McGeer [66], persistent neuroinflammatory processes, however, might be part of the series of events leading to the progressive neuronal damage seen in many CNS pathologies, including ischemic stroke, a cerebrovascular disorder [67], and neurodegenerative disorders such as Parkinson’s disease (PD) and Alzheimer’s disease (AD) [68,69]. AD is primarily caused by chemical nocive exposures and air pollution, which are primarily experienced by vulnerable individuals during their formative years. These exposures result in high levels of frontal tau hyperphosphorylation with pre-tangle material and diffuse plaques of amyloid-β [70,71,72]. In the absence of established risk factors for cognitive and neurological abnormalities, children show MRI prefrontal white matter hyperintensities (WMH) and severe selective impairment in attention, short-term memory, and learning capacity [73]. According to Calderón-Garcidueñas and colleagues [70], monochorionic–monoamniotic (MCMA) children showed an early brain imbalance in genes related to oxidative stress, inflammation, innate and adaptive immunological responses, cell proliferation, and apoptosis. Cognitive impairment and the pathogenesis of neurodegenerative states are clearly influenced by neuroinflammation, endothelial activation, endothelial cell hyperplasia, the attachment of white blood cells to the endothelial damaged walls with the reduction of the lumen vessel, high plasmatic concentrations of endothelin-1, and the breakdown of the blood–brain barrier [74,75]. Consequently, it has been suggested that using non-steroidal anti-inflammatory drugs (like ibuprofen) could postpone or even stop the onset of these neurodegenerative disorders [76,77]. Additionally, epidemiologic studies have shown that anti-inflammatory drug users had a lower risk of developing AD [78]. But currently available medications mostly address the signs and symptoms of these neurodegenerative diseases rather than stopping the underlying neuronal loss. As a result, there is a need to create new treatments that can stop the pathological loss of certain neuronal populations that causes these disorders from progressing [79]. Here, we have obtained experimental results that pointed out the importance of controlling the expression of CD11b in leukocytes since their levels increase significantly during the inflammation process. In consequence, our results demonstrated that the cocoa-enriched extract diet reduces notably the pathological effect of CD11b expression pattern when inflammation is induced, being at low levels at the main affected brain regions such as the frontal cortex layers, dentate gyrus, thalamus, and hypothalamus. Our present results are in line with prior research on rodents that have demonstrated that dark chocolate reduces dorsal vagal complex inflammation in mice exposed to MCMA [80]. Therefore, positive effects such as angiogenesis, neurogenesis in learning and memory regions, improved cognition, improved endothelial function, a significant decrease in oxidative stress, the production of pro-inflammatory cytokines and eicosanoids, and platelet activation are among the benefits of cocoa and dark chocolate [81,82,83,84,85,86,87,88]. In a meta-analysis evaluation of more than a thousand participants looking at the short-term effects of flavonoid-rich cocoa, Shrime and colleagues [89] found that adults’ blood pressure, insulin resistance, lipid profiles, and flow-mediated vascular dilatation were all significantly improved by chocolate intake.

Cocoa, berries, almonds, pomegranate juice, oranges, apples, and sweet cherries are some of the foods highest in polyphenols [90]. When it comes to memory and frontal executive functions, such as processing speed and attention, these foods seem to have the most positive effects. It is conceivable that several polyphenols collaborate with one another and other nutrients to provide cognitive benefits. In particular, flavonoids are abundant in cocoa, which is made from the dried and fermented seeds of *Theobroma cacao*. Cocoa is particularly high in catechin, other oligomeric procyanidins, and the flavan-3-ol epicatechin. Epicatechins appear to penetrate the blood–brain barrier in animals and may have direct effects on the brain in addition to being bioavailable in humans. Through enhanced neurogenesis, particularly the proliferation and survival of new hippocampus neurons, and an increase in synaptic development through the activation of BDNF, flavonoids derived from cocoa have been linked to improving neuroplasticity [91,92,93]. Recently, flavonoids are being investigated extensively as possible nutraceuticals with neuroprotective properties. It is interesting to note that cocoa beans, which contain significant amounts of flavan-3-ols and their derivatives, have been identified as a primary source of antioxidant flavonoids. Flavan-3-ols, a kind of flavonoid found in tea, chocolate, pome fruits, grapes, and berries, have been linked to several positive effects on brain areas involved in memory formation and multiple risk factors for cognitive performance. Thus, current research has concentrated on the benefits of chocolate and cocoa rich in flavonoids on cognitive performance and risk factors for cerebrovascular disease. Preclinical and clinical research point to endothelial vascular effects and anti-inflammatory qualities as two possible causes of these beneficial effects on cognitive function. A study on the effects of polyphenols on mild cognitive impairment in ninety-nine elderly people (aged 64–82) revealed that a high intake of cocoa flavan-3-ol (993 mg/day, 8 weeks) improved cognitive function as measured by the Mini-Mental State Examination (MMSE), the Trail Making Test (TMT) A and B, and the Verbal Fluency Test (VFT) [84]. Similarly, hippocampal dentate gyrus function was improved in older people by supplementing with cocoa flavonoids (900 mg/day for three months) in a healthy cohort of 34 male and female participants (aged 50 to 69). The authors suggested that a particular increase in capillary density in the dentate gyrus may be responsible for this strategy of preventing age-related cognitive loss [94]. Flavan-3-ols decreased the risk of Alzheimer’s disease and similar dementias due to decreased brain degradation [95], decreased cognitive deterioration rates [96], enhanced verbal memory, and long-term linguistic proficiency [97];pre- and post-assessment using fMRI and cognitive testing revealed enhanced dentate gyrus function associated with delayed age-related cognitive decline [94], greater cognitive performance, and improved brain oxygenation during intense cognitive demands [98]. Catechins decreased cognitive dysfunction and increased cognitive performance [99].

The central nervous system’s sentinel agents are microglial cells [100]. Although they play a role in neuroprotection, they are also strongly linked to the neurodegeneration of the aging brain [101,102]. Nitric oxide (NO) and cytokines are pro-inflammatory substances released by hyperactivated microglia that play a crucial role in inducing neuroinflammatory responses linked to neurodegenerative disorders [103,104]. This work investigated the potential anti-inflammatory and neuroprotective effects of bromelain, the pineapple-derived extract, and spirulina phytochemical contents on primary microglia through regulation of microglial activation [105,106,107]. The present study demonstrates that the combined extract of cocoa, pineapple, and spirulina reduces dramatically the adverse effects of neuroinflammation as shown by specific biomarker analysis in mice model’s brain. In particular, researchers have recently focused on the therapeutic activity of bromelain, a major protease enzyme found in pineapple (*Ananas comosus*), as an anti-inflammatory agent. It also has other effects, such as modulating tumor growth, healing wounds, and treating arthritis, episiotomy, and muscular pain [108,109]. Furthermore, investigations on acute and inflammatory pain in humans and animals have shown that bromelain has antinociceptive properties [110]. However, studies have demonstrated that spirulina has the following beneficial effects: it is hypolipemic, antihypertensive, antidiabetic, neuroprotective, antianemic, anticarcinogenic, hepatoprotective, and an immunomodulatory, antibacterial, and antiviral agent [111,112,113]. By controlling important cytokines like IL-1β, IL-2, IL-4, IL-6, IL-10, TNF-α, and IFN-γ, spirulina can promote phagocytosis in macrophages [114,115], as well as generate cellular and humoral adaptive immunity [114,116,117]. Although cyclooxygenase-2 (COX-2) activity may be inhibited by spirulina (Reddy), the protein extract of spirulina also exhibits chelating capabilities, lowers DNA damage and lipid peroxidation, and scavenges free radicals [118,119]. Spirulina’s phycocyanin concentration, which in turn attributes its antioxidant activity to phycocyanobilin [120], may be responsible for its antioxidant and free radical-scavenging qualities. Taken all together, the present study’s findings regarding inflammation biomarkers in mouse models’ brains provide some insight into the creation of novel natural medications, particularly natural bioactive products, that can treat neurological and neurodegenerative diseases by reversing inflammatory imbalance and the ensuing cell damage without endangering neurons. We have created a rich source of antioxidant and anti-inflammatory compound by combining the special bioactive properties of cocoa, pineapple, and spirulina. This compound can compensate for vitamin nutritional deficiencies and support the development of the nervous system and physiologic brain functions. It can also promote a beneficial immune response. However, despite the numerous and encouraging scientific evidence both in vitro and in vivo, additional studies are needed to clarify the mechanisms of action of each one of the cocoa-enriched extract components.

## 5. Conclusions

Preclinical and clinical evidence from research studies during the last decade strongly demonstrates that plant-based dietary patterns [121,122] are a useful and a practical approach to preventing chronic diseases. Since oxidative stress, inflammation, and vascular damage are among the numerous pathophysiological pathways that neurodegenerative disorders and cardiovascular disease share, it makes sense to assume that plant-based diets can also slow the decline in cognitive function. In the present study we have focused on the potential effects of cocoa against inflammation, enhanced by synergic polyphenol-rich compounds such as spirulina and pineapple. Certain cognitive domains, most notably frontal executive skills including attention, processing speed, and memory encoding, consolidation, and retrieval, appear to be the areas where polyphenols have a protective impact. Increased cerebral blood flow, decreased oxidative stress and neuroinflammation, enhanced neurogenesis, and improved neuroplasticity are potential processes underpinning these neurocognitive advantages [123,124]. Here, we have demonstrated that a plant-based cocktail diet supplemmentation (cocoa, spirulina, and pineapple) might prevent cognitive decline by modifying neuroinflammation biomarkers in mice. These mechanisms have all been connected to cognitive processes that are particularly related to memory and executive function, as reported previously [124,125]. The present findings seem novel since they show that cocoa enriched extract bioactive properties and components [126,127,128,129,130,131,132] appears to influence, either directly or indirectly, signal transduction pathways implicated in brain inflammation markers. This suggests that cocoa extract acts as a neuroprotection agent by preventing against oxidative stress-related neurodegenerative diseases. Moreover, by interfering with redox-regulated pathways, cacao’s antioxidant components can reduce the breakdown of tryptophan, the generation of serotonin, and the concentration of inflammatory markers. However, although many bioactive components are still present in processed cocoa products like chocolate or cacao, the health effects of these products may differ from those of raw cocoa extracts since they are typically consumed within a complex food matrix.

## Figures and Tables

**Table 1:** Experimental protocol table showing mice group A treated with a poor diet (PD) and mice group B treated with cocoa extract supplementation (CED) on the poor diet, during 5 weeks.

	Weeks of Treatment				
	1	2	3	4	5
Gr A (Poor Diet)	PD	PD	PD	PD	PD
Gr B (Poor Diet/Cocoa extract Diet)	PD + CED	PD + CED	PD + CED	PD + CED	PD + CED

**Table 2:** Analyzed parameters carried out on the experimental groups to address the effectiveness of the cocoa-enriched extract on the state of hypo-vitaminosis and neuroinflammation. *p* value of 0.05 or less is considered statistically significant. HCT: Hematocrit; HGB: Hemoglobin; MCH: Mean Corpuscular Hemoglobin; MCHC: Mean Corpuscular Hemoglobin Concentration; MCV: Mean Corpuscular Volume; MPV: Mean Platelet Volume; PCR: C-reactive protein; PCT: Platelet crit; PLT: Platelets; RBC: Erythrocytes (red blood cells); VB12: Vitamin B12; VB6: Vitamin B6; VB9: Vitamin B9 (folate); WBC: Leukocytes (white blood cells).

PARAMETER	UNITS	GROUP	N	MEAN	STANDARD DEVIATION	STANDARD ERROR	*t*-TEST Sig (*p*)
**IMMUNE/ENERGETIC/ANTI-ANEMIC EFFECT**							
**WBC**	× 10^9^/L	CONTROL	3	0.5600	0.17349	0.1017	*p* > 0.05
		TREATMENT	4	3.3725	2.14304	1.07152	
**RBC**	× 10^12^/L	CONTROL	3	9.3500	0.32047	0.18502	*p* > 0.05
		TREATMENT	4	8.2400	1.45788	0.72894	
**HGB**	g/dL	CONTROL	3	13.8667	0.47258	0.27285	*p* > 0.05
		TREATMENT	4	11.9000	1.79444	0.89722	
**HCT**	%	CONTROL	3	45.7333	2.84488	1.64249	*p* > 0.05
		TREATMENT	4	40.5250	5.77430	2.88715	
**MCV**	fL	CONTROL	3	48.9000	1.34536	0.77675	*p* > 0.05
		TREATMENT	4	49.5250	3.01703	1.50852	
**MCH**	pg	CONTROL	3	14.8000	0.1000	0.05774	*p* > 0.05
		TREATMENT	4	14.5000	0.46904	0.23452	
**MCHC**	g/dL	CONTROL	3	30.3667	0.95044	0.54874	*p* > 0.05
		TREATMENT	4	29.3500	1.56098	0.78049	
**PLT**	× 10^9^/L	CONTROL	3	1773.3333	130.12430	75.12730	*p* = 0.025
		TREATMENT	4	1313.5000	67.81593	33.90796	
**MPV**	fL	CONTROL	3	5.1333	0.15275	0.08819	*p* = 0.023
		TREATMENT	4	5.6500	0.23805	0.11902	
**PCT**	%	CONTROL	3	0.5520	0.05603	0.03235	*p* = 0.002
		TREATMENT	4	0.7415	0.03215	0.01608	
**ANTI-INFLAMMATORY EFFECT**							
**PCR**	mg/L	CONTROL	2	0.6000	0.28284	0.20000	*p* > 0.05
		TREATMENT	5	0.4600	0.20736	0.09274	
**NUTRITIONAL EFFECT/VITAMINS**							
**VB6**	ng/mL	CONTROL	3	7.0393	2.2942	1.3246	*p* > 0.05
		TREATMENT	4	16.5985	5.4066	2.7033	
**VB9**	ng/mL	CONTROL	3	20.4733	5.4484	3.1456	*p* > 0.05
		TREATMENT	5	27.4142	8.7669	3.9207	
**VB12**	pg/mL	CONTROL	3	7675.8333	2432.8280	1404.5939	*p* > 0.05
		TREATMENT	4	9897.500	6542.4444	3271.2222	

## Data Availability

The original contributions presented in this study are included in the article/Supplementary Materials. Further inquiries can be directed to the corresponding author(s).

## Supplementary Materials

The following supporting information can be downloaded at https://www.mdpi.com/article/10.3390/neurolint17040047/s1. Table S1: Cocoa-derived extract composition; Table S2: The nutritional content of cocoa in percentage per 100 g; Table S3: Compounds found in Cocoa and their formulas, structures and functions; Table S4: The nutritional content of pineapple pulp in percentage per 100 g; Table S5: The nutritional content of spirulina in percentage per 100 g.
