# Supplementary material for: Preventive Role of Cocoa-Enriched Extract Against Neuroinflammation in Mice
Source: Neurol Int. 2025 Mar 24;17(4):47. doi: 10.3390/neurolint17040047 (PMC12029631; doi:10.3390/neurolint17040047)
Supplement: Supplementary file 1 [file neurolint-17-00047-s001.zip › neurolint-3500170-supplementary.pdf]

SUPLEMENTARY MATERIAL

Table S1. Cocoa-derived extract composition:

COCOA	*Theobroma cacao*
PINEAPPLE	*Ananas comosus*
SPIRULINA	*Arthrospira Platensis*

COCOA (*Theobroma cacao*)

Reference: Edo G I, Samuel P O, Oloni G O, et al. Review on the Biological and Bioactive components of Cocoa (Theobroma Cacao). Insight on Food, Health and Nutrition. Natural Resources for Human Health. 2023;3(4):426-448. doi:10.53365/nrfhh/174302.

Table S2. The nutritional content of cocoa in percentage per 100g.

**Macronutrients**	**Percentage**
Protein	15-21%
Carbohydrates	~15%
Lipids	10-25%
Fiber	25-40%
**Micronutrients**	**Per 100g**
Vitamin A(Retinol)	<0.2mg
Vitamin E(Tocopherol)	2.5mg
Vitamin B1(Thiamine)	0.3mg
Vitamin B1(Riboflavin)	0.4mg
Vitamin B3(Niacin)	0.7mg
**MINERALS**	**Per 100g**
Sodium (Na)	0.03g
Potassium (k)	4.3g
Calcium (ca)	151mg
Phosphorus (P)	700mg
Iron (Fe)	26mg
Magnesium (mg)	555mg
Copper (cu)	5mg.

Bioactive Compounds of Cocoa

Phenolics

Among the many bioactive substances included in cocoa are phenolics, which include procyanidins, flavonoids, and catechins. These phenolic chemicals contribute significantly to the antioxidant qualities of cocoa in addition to giving it its unique flavor and scent (Melo et al., 2021). Because of its antioxidant properties, cocoa phenolics assist the body fight off dangerous free radicals, which lessens oxidative stress and may lower the chance of developing a number of chronic illnesses, including cancer and heart problems. Moreover, Sorrenti and colleagues (2020) have linked these phenolic compounds to anti-inflammatory properties.

Phenolic Composition Analysis of Cocoa Varieties

Cocoa beans naturally contain phenolic compounds, such as flavonoids and polyphenols, which are beneficial substances. According to Martin et al. (2021), they are crucial in determining the nutritional value and sensory attributes of cocoa and chocolate. According to Urbańska and Kowalska (2019), these chemicals have an impact on the flavor profile as well as the antioxidant characteristics that have drawn attention to cocoa and dark chocolate due to their possible health advantages. This section examines the phenolic makeup of many cocoa cultivars, each distinguished by a particular flavor profile and set of characteristics. By conducting a thorough examination and measurement of these phenolic chemicals in cocoa mucilage and cocoa bean shell, we hope to gain important understanding of the distinctions and parallels between these types.

Table S3. Compounds found in Cocoa and their formulas, structures and functions.

S/N	COMPOUND	FORMULA	FUNCTIONS/USES	
1	flavanols	C15H14O2	They have anti-inflammatory, cardioprotective, anti-cancer, hypolipidemic, anti-diabetic, anti-microbial, and anticancer properties.	(Urbańska & Kowalska, 2019)
2	Anthocyanidins	C15H11O+	I. They mostly serve the purpose of coloring plant parts with red, blue, and purple shades, among other colors. ii. They help to avoid diabetes and obesity. III.Anthocyanins have been proved to have the ability to modify cognitive and motor performance, improve memory, and play a part in delaying the effects of aging on cerebral function.	(Jimmy & Jose, 2011).
3	Proanthocyanidins	C31H28O12	I. They boost blood vessel strength in those who have diabetes (increased blood sugar level) and high blood pressure (hypertension). ii. They helps in the prevention of cancer iii. They also assist in the treatment of nutritional deficiencies.	(Liang et al., 2021)
4	Caffeine	C8H10N4O2	It helps the brain and neurological system to be more active. it also causes a greater flow of hormones like cortisol and adrenaline throughout	(Pucciarelli, 2013)

S/N	COMPOUND	FORMULA	FUNCTIONS/USES	
			the body. Caffeine can also help you feel awake and alert in moderation.	
5	Theobromine	C7H8N4O2	It functions as a diuretic, a vasodilator, and a cardiac stimulant. Additionally, it might help in the treatment of fatigue and orthostatic hypotension.	(Feo et al., 2020)
6	Methylxanthines	C6H6N4O2	They relax the smooth muscles of the bronchi, esophagus, and gastroesophageal sphincter.	(Ludovici et al., 2017)
7	Catechin	C15H14O6	They assist control vascular tone by triggering endothelial nitric oxide. Due to the anti-inflammatory effects of the capillaries and the suppression of vascular cell growth factors involved in atherosclerosis, it also contributes significantly to the prevention of the progression of atherosclerosis.	(Mendoza-Meneses, Feregrino-Pérez, & Gutiérrez-Antonio, 2021)
8	Procyanidins	C30H26O13	They protect the circulatory and heart systems. They might act as antioxidants and prevent nitrosamine formation. They might guard against their effects in healthy cells. Together, they and vitamin C reduce the risk of breast cancer.	(Balentić et al., 2018)

Bibliography:

- Melo TS, Pires TC, Engelmann JVP, Monteiro ALO, Maciel LF, Bispo EDS. Evaluation of the content of bioactive compounds in cocoa beans during the fermentation process. J Food Sci Technol. 2021 May;58(5):1947-1957.
- Sorrenti V, Ali S, Mancin L, Davinelli S, Paoli A, Scapagnini G. Cocoa Polyphenols and Gut Microbiota Interplay: Bioavailability, Prebiotic Effect, and Impact on Human Health. Nutrients. 2020 Jun 27;12(7):1908.
- Martin MÁ, Ramos S. Impact of cocoa flavanols on human health. Food Chem Toxicol. 2021 May;151:112121.
- Urbańska B, Kowalska J. Comparison of the Total Polyphenol Content and Antioxidant Activity of Chocolate Obtained from Roasted and Unroasted Cocoa Beans from Different Regions of the World. Antioxidants (Basel). 2019 Aug 6;8(8):283.

PINEAPPLE (*Ananas comosus*)

Reference: Maimunah Mohd Ali, Norhashila Hashim, Samsuzana Abd Aziz, Ola Lasekan. Pineapple (Ananas comosus): A comprehensive review of nutritional values, volatile compounds, health benefits, and potential food products. Food Research International, Volume 137, November 2020, 109675.

Table S4. The nutritional content of pineapple pulp in percentage per 100g.

**Macronutrients**	**Percentage**
Protein	1%
Carbohydrates	12%
Lipids	1%
Fiber	5%
**Micronutrients**	**Percentage**
Provitamin A Beta-carotene (µg)	17 - 82
Vitamin A equivalent (µg)	2,83 - 14
Vitamin B1 (mg)	0,05 - 0,11
Vitamin B2 (mg)	0,01 - 0,052
Vitamin B3 (mg)	0,17 - 1,49
Vitamin B5 (mg)	0,05 - 0,45
Vitamin B6 (mg)	0,01 - 0,15
Vitamin B9 (µg)	8 - 36
Vitamin C (mg)	7,90 - 70,70
Vitamin E (mg)	0,02 - 0,10
Vitamin K1 (µg)	0,20 - 1,20
**Minerals**	**Per 100g**
Sodium (Na)	1mg
Potassium (k)	140mg
Calcium (ca)	8mg
Phosphorus (P)	9mg
Iron (Fe)	0,1mg
Manganese (mg)	0,9mg
Copper (cu)	0,06mg.

Note: ANSES. Ciqual Table, Nutritional composition of pineapples, 2020. Source: (ANSES, 2020).

SPIRULINA (*Arthrospira Platensis*)

Reference: Koli DK, Rudra SG, Bhowmik A, Pabbi S. Nutritional, Functional, Textural and Sensory Evaluation of Spirulina Enriched Green Pasta: A Potential Dietary and Health Supplement. Foods. 2022 Mar 28;11(7):979. doi: 10.3390/foods11070979. PMID: 35407065; PMCID: PMC8997815.

Table S5: The nutritional content of spirulina in percentage per 100g

Macronutrients.	Percentage
Protein	66%
Carbohydrates	22%
Lipids	7%
Fiber	9%
**Micronutrients**	**Per 100g**
Vitamin A	29µg
Retinol	0µg
beta-Carotene	342µg
Thiamine	2.4mg
Riboflavin	3.7mg
Niacin	28NE
Pantothenic acid	3.5mg
Vitamin B6	0.36mg
Folic acid	94µg
Vitamin B12	0µg
Vitamin C	10mg
Vitamin D	0µg
Vitamin E	5mg
Vitamin K	26µg
**Minerals**	**Per 100g**
Histidine	1.1g
Isoleucine	3.2g
Leucine	4.9g
Lysine	3g
Methionine	1.1g
Phenylalanine	2.8g
Threonine	3g
Tryptophan	0.93g
Valine	3.5g
